# Identification of Differentially Expressed Genes and Signaling Pathways in Acute Myocardial Infarction Based on Integrated Bioinformatics Analysis

**DOI:** 10.1155/2019/8490707

**Published:** 2019-08-01

**Authors:** Da-Qiu Chen, Xiang-Sheng Kong, Xue-Bin Shen, Mao-Zhi Huang, Jian-Ping Zheng, Jing Sun, Shang-Hua Xu

**Affiliations:** ^1^Department of Cardiology, Affiliated Nanping First Hospital, Fujian Medical University, Nanping 353000, Fujian Province, China; ^2^Department of Medical Laboratory Medicine, Affiliated Nanping First Hospital, Fujian Medical University, Nanping 353000, Fujian Province, China; ^3^Department of Cardiology, Affiliated Nanping First Hospital, Fujian Medical University, Zhongshan Road 317th, Nanping 353000, China

## Abstract

**Background:**

Acute myocardial infarction (AMI) is a common disease with high morbidity and mortality around the world. The aim of this research was to determine the differentially expressed genes (DEGs), which may serve as potential therapeutic targets or new biomarkers in AMI.

**Methods:**

From the Gene Expression Omnibus (GEO) database, three gene expression profiles (GSE775, GSE19322, and GSE97494) were downloaded. To identify the DEGs, integrated bioinformatics analysis and robust rank aggregation (RRA) method were applied. These DEGs were performed through Gene Ontology (GO) and Kyoto Encyclopedia of Genes and Genomes (KEGG) pathway analyses by using Clusterprofiler package. In order to explore the correlation between these DEGs, the interaction network of protein-protein internet (PPI) was constructed using the STRING database. Utilizing the MCODE plug-in of Cytoscape, the module analysis was performed. Utilizing the cytoHubba plug-in, the hub genes were screened out.

**Results:**

57 DEGs in total were identified, including 2 down- and 55 upregulated genes. These DEGs were mainly enriched in cytokine-cytokine receptor interaction, chemokine signaling pathway, TNF signaling pathway, and so on. The module analysis filtered out 18 key genes, including* Cxcl5, Arg1, Cxcl1, Spp1, Selp, Ptx3, Tnfaip6, Mmp8, Serpine1, Ptgs2, Il6, Il1r2, Il1b, Ccl3, Ccr1, Hmox1, Cxcl2,* and* Ccl2*.* Ccr1* was the most fundamental gene in PPI network. 4 hub genes in total were identified, including* Cxcl1, Cxcl2, Cxcl5, *and* Mmp8*.

**Conclusion:**

This study may provide credible molecular biomarkers in terms of screening, diagnosis, and prognosis for AMI. Meanwhile, it also serves as a basis for exploring new therapeutic target for AMI.

## 1. Introduction

Acute myocardial infarction (AMI), which represents the main public health issue around the world, is a common cardiac emergency with substantial morbidity and mortality. In the last two or three decades, although a downtrend of AMI has been observed because of the economic development and advances in medical science, its morbidity is still very high at about 44.57 in 100,000 people in China in 2013 [1]. Besides, the death rate of AMI was estimated to increase by 5.6 times from 1987 to 2014 [2]. Therefore, it is growing important for AMI to develop an early diagnosis and proper treatment strategy to prevent the occurrence of sudden mortality.

Fortunately, with the development of gene chip technique, more and more gene expression spectra were tested by gene chip technique in cardiovascular clinic and study. Microarray analysis was widely used in peripheral blood of patients with myocardial infarction [3] and the myocardium of mice [4]. Through microarray analysis, the potential genes associated with AMI will be obtained. For example, through the microarray analysis of GSE48060, Yuan Gao et al. [3] found that the* MAX, BCL3, NCOA7, CCL5*, and* GTF3C2 *might play a key role in AMI development, which provided valuable reference for future research. Many studies have found that early growth response factor 1 (EGR1) induces myocardial injury after AMI. Using bioinformatics analysis, Pan et al. [5] found that miR-146a can regulate the expression of EGR1. It offers help as treatment for AMI. Under many stringent states, including ischemia reperfusion, Heat shock proteins (Hsps) are produced. Novo G et al. [6] expounded the clinical significance and pathogenetic role of Hsp60 and HO-1 in AMI using bioinformatics analysis. Heart failure (HF) is a common complication after AMI. Qian C et al. [7] found that the DEGs, including* FOS, THBS1, CXCL8, *and* ITGA2B* from the microarray data of GSE59867, may play a vital role in the occurrence and development of HF after AMI. In recent years, integrated bioinformatics analysis method is heavily used in cancer. For example, utilizing integrated bioinformatics analysis method, Guangwei et al. [8] reported the novel therapeutic targets for colorectal neoplasms. However, integrated bioinformatics analysis method is rarely employed in cardiovascular disease.

In this research, three gene expression datasets, including GSE775, GSE19322, and GSE97494, were downloaded from the GEO database. These datasets were screened to identify the DEGs in each dataset. Next, using the RRA approach [9], a total of 57 DEGs, including 2 down- and 55 upregulated genes, were identified. Using Clusterprofiler [10], GO and KEGG analyses were performed, respectively. It was obviously shown that these DEGs were enriched in AMI-related functions and pathways. Then the PPI network was established by using the STRING database. The module analysis filtered out 18 key genes, including* Cxcl5, Arg1, Cxcl1, Spp1, Selp, Ptx3, Tnfaip6, Mmp8, Serpine1, Ptgs2, Il6, Il1r2, Il1b, Ccl3, Ccr1, Hmox1, Cxcl2,* and* Ccl2*.* Ccr1* was the most fundamental gene in PPI network. 4 hub genes in total were identified, including* Cxcl1, Cxcl2, Cxcl5, *and* Mmp8.* Our result may provide a novel pathway for diagnosis and treatment of the AMI in the future.

## 2. Methods

### 2.1. Affymetrix Macroarray Data

Utilizing the keywords “myocardial infarction,” we screened the GEO database. Three GEO datasets were found, including GSE775 contributed by Schinke et al., GSE19322 contributed by Hunt et al., and GSE97494 contributed by Chikata et al. These gene expression profiles of AMI were downloaded based on GPL81 platform of Affymetrix Murine Genome U74A Version 2 Array, GPL339 platform of Affymetrix Mouse Expression 430A Array, and GPL6246 platform of Affymetrix Mouse Gene 1.0 ST Array, respectively. There were 18 samples that were from the region between the LAD artery and the apex of the mice, 9 mice within 24 hours after AMI and 9 sham-operated mice within 24 hours. Detailed information about the datasets is listed in Table 1. Through the R software package, the download files were handled.

### 2.2. Screening for DEGs

In order to find out DEGs of each GEO dataset, utilizing the R software and annotation package, the platform and series matrix file(s) were converted. These DEGs in AMI and sham operation group samples were analyzed by utilizing the limma package [11] in R. Log2(fold change) (log2FC) > 1 and a corrected *p* value < 0.05 were used as the cut-off criteria of DEGs samples.

### 2.3. Integration of Microarray Data

Through limma packet analysis, we obtained the list of DEGs of the three microarray datasets. The list of down- and upregulated genes in the microarray data was saved. Subsequently, using the RRA approach, the comparison of multiple ranked gene lists was performed.

### 2.4. GO and KEGG Pathway Enrichment Analyses

Biological functions of the DEGs obtained from the integration of microarray data were explored with GO analysis using Clusterprofiler which is an R package utilized to compare the biological themes among gene clusters. Similarly, in order to identify the enrichment signaling pathways of DEGs, KEGG pathway analysis was performed by utilizing the Clusterprofiler package. A corrected* p* < 0.05 was the cut-off criterion.

### 2.5. PPI Network Integration, Modules Analysis, and Selection of Hub Genes

In order to identify the interaction between PPI, the PPI network was built using the STRING (version 11) online database. The highest confidence of the argument of interactions was set at >0.4. To draw an interaction of DEGs, the Cytoscape (version 3.6.1) software was used to visualize and analyse the PPI network. In order to find modules of the whole network, the Molecular Complex Detection (MCODE) plug-in of the Cytoscape software was applied. The hub genes were identified by using the plug-in cytoHubba [12] of the Cytoscape software, including Density of Maximum Neighborhood Component (DMNC) and Maximal Clique Centrality (MCC).

## 3. Results

### 3.1. Identification of DEGs in GSE775, GSE19322, and GSE97494

**Figure 1:**
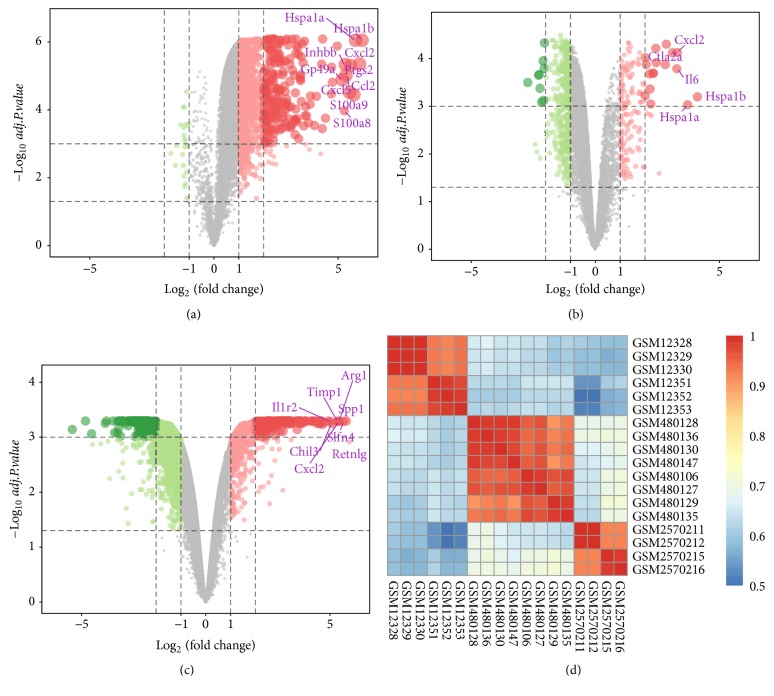
Volcano plot of gene expression profile data in AMI samples and sham-operation ones and correlation coefficient analysis diagram. Notes: (a) volcano plot of GSE775, (b) volcano plot of GSE19322, and (c) volcano plot of GSE97494. The red, green, and gray points represent upregulated genes, downregulated genes, and nondifferentially expressed genes, respectively. They are screened on the basis of log2FC >1.0 and a corrected p <0.05. (d) Correlation coefficient analysis diagram. Each column and row, respectively, represents one sample. Red represents strong correlation between samples and grey represents weak correlation between samples. Different shades of colors indicate the different correlation degree. Abbreviation: AMI, acute myocardial infarction; DEGs, differentially expressed genes; FC, fold change.

**Figure 2:**
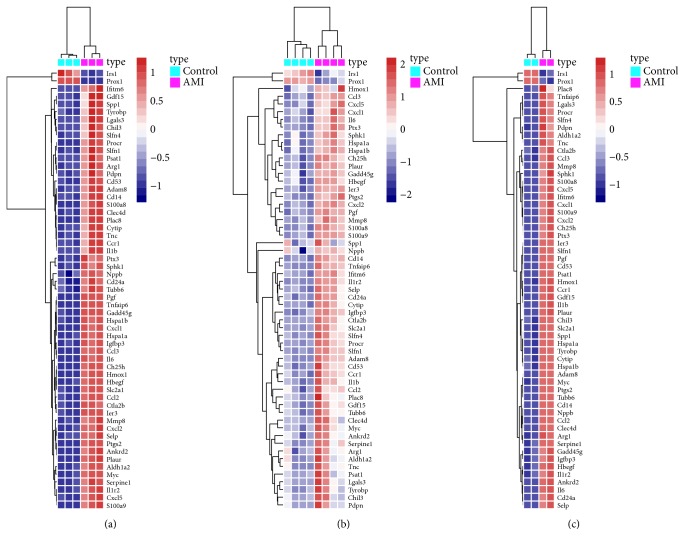
Hierarchical clustering heatmap of DEGs, which was screened on the basis of log2FC >1.0 and a corrected p <0.05. Notes: (a) GSE775 data, (b) GSE19322 data, and (c) GSE97494 data. Red represents that the expression of genes is relatively upregulated. Blue represents that the expression of genes is relatively downregulated. Gray represents the expression of genes without significant changes. Abbreviation: DEGs, differentially expressed genes; FC, fold change.

Three expression microarray datasets, including GSE775, GSE19322, and GSE97494, were used to perform background correction and quartile data normalization by the limma package. Meanwhile, using the limma package (log2FC >1, corrected* p* <0.05), the GSE775 dataset was screened and 2149 DEGs were obtained, including 23 down- and 2126 upregulated genes. Using the same methodology, 597 DEGs were obtained from the GSE19322 dataset, including 446 down- and 151 upregulated genes, and 4534 DEGs were confirmed from the GSE97494 dataset, including 3879 down- and 655 upregulated genes. Many DEGs in two sets of sample data of each microarray, three microarrays in total, are shown in Figures 1(a)–1(c), also known as the volcano plots of DEGs. In order to evaluate the biological repeatability, we drew an association diagram, which indicated that the biological repeatability of the sample was well, as shown in Figure 1(d). Three cluster heatmaps of the 57 DEGs in each microarray are shown in Figures 2(a)–2(c).

### 3.2. Identification of DEGs in AMI Utilizing Integrated Bioinformatics Analysis

**Figure 3:**
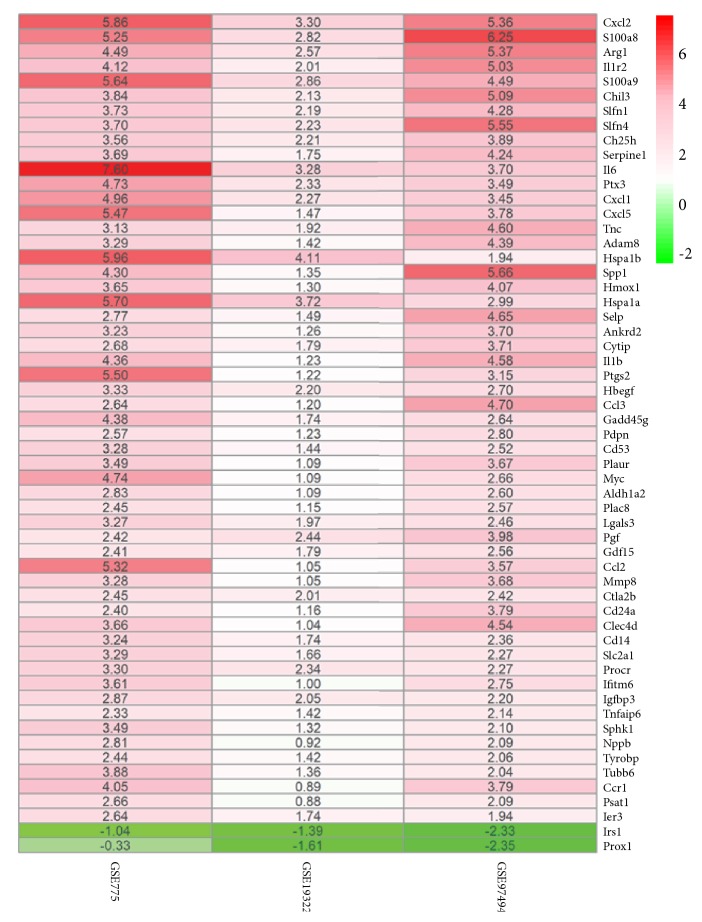
The heatmap of differentially expressed genes. Notes: each column and row represents one dataset and one gene, respectively. Red and green represent logFC >0 and logFC <0, respectively. The logFC values are shown in each rectangle. The gradual color ranged from green to red represents the changing process from downregulation to upregulation. Abbreviation: FC, fold change.

Using the RRA method according to Log2FC >1 and a corrected* p* <0.05, the list of DEGs of the three microarray datasets were analyzed. A total of 57 DEGs were determined by rank analysis, including 2 down- and 55 upregulated genes, as shown in Table 2. The heatmap of the 57 DEGs was drawn by heatmap package, which is shown in Figure 3.

### 3.3. GO Analysis of DEGs

**Figure 4:**
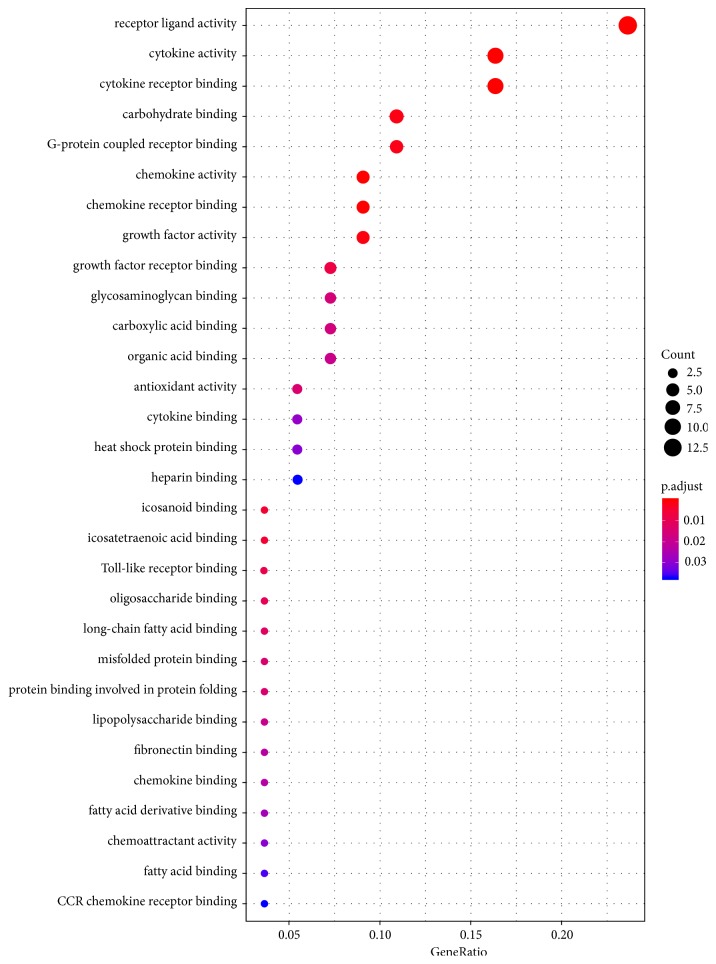
GO enrichment analyses of DEGs in AMI. Notes: X-axis indicates the percentage that each functional group gene, respectively, accounts for the total genes. Y-axis represents different functional groups (also named as different GO terms). The size of the dot indicates the number of genes in different functional groups, and the color of the dot reflects the different* p*-value range. The bigger the gene count, the bigger the dot size is. The gradual color ranged from blue to red represents the changing process of* p*-value from big to small value. Abbreviations: AMI, acute myocardial infarction; DEGs, differentially expressed genes; GO, gene ontology.

Using Clusterprofiler package, biological annotation of the DEGs obtained by RRA approach was performed. The down- and upregulated genes with *p* value <0.05 were obtained from GO functional enrichment. From GO functional enrichment analysis, we identified that these DEGs were mainly enriched in the following functional categories, including receptor ligand activity, cytokine activity, cytokine receptor binding, G-protein coupled receptor binding, carbohydrate binding, chemokine activity, and chemokine receptor binding. GO analyses are shown in Figure 4. Meaningful results of the GO analysis of DEGs in AMI are listed in Table 3.

### 3.4. KEGG Pathway Analysis of DEGs

**Figure 5:**
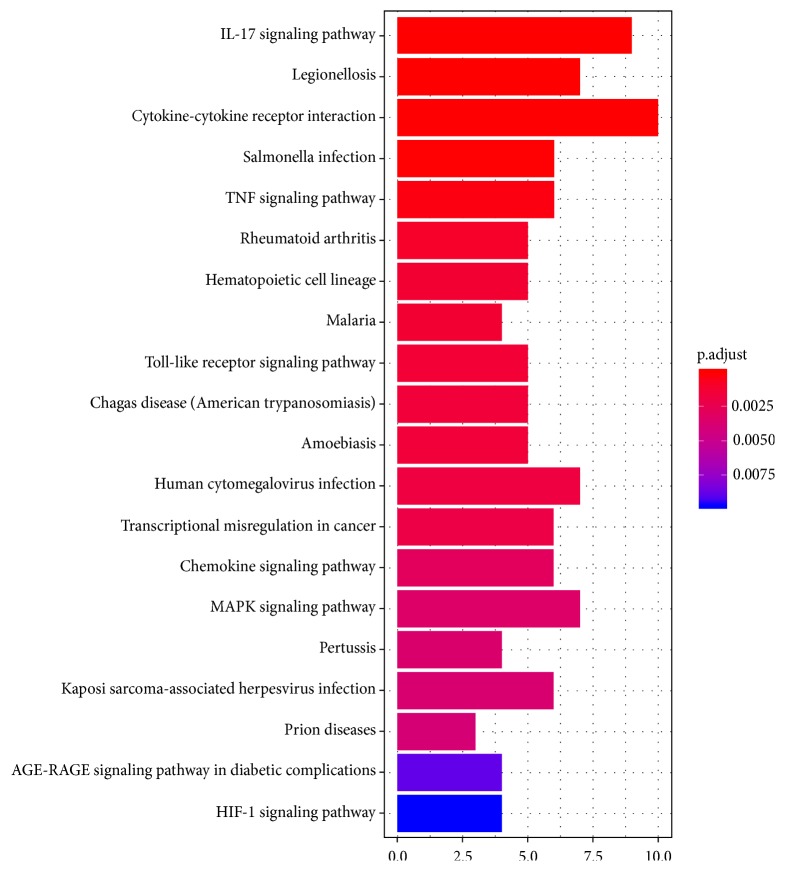
Top 20 KEGG pathway enrichment analyses of DEGs in AMI. Notes: X-axis indicates gene count; Y-axis represents different pathways. The column color reflects* p*-value: red represents the smallest value; blue represents the biggest value. The gradual color ranged from blue to red represents the changing process of* p*-value from big to small value. Abbreviations: AMI, acute myocardial infarction; DEGs, differentially expressed genes; KEGG, Kyoto Encyclopedia of Genes and Genomes.

Top 20 KEGG pathway analyses of DEGs are shown in Table 4 and Figure 5. Table 4 shows that these DEGs were primarily enriched in the cytokine-cytokine receptor interaction, Chemokine signaling pathway, TNF signaling pathway, and so on.

### 3.5. Establishing the PPI Network, Conducting Modules Analysis, and Selection of Hub Genes

**Figure 6:**
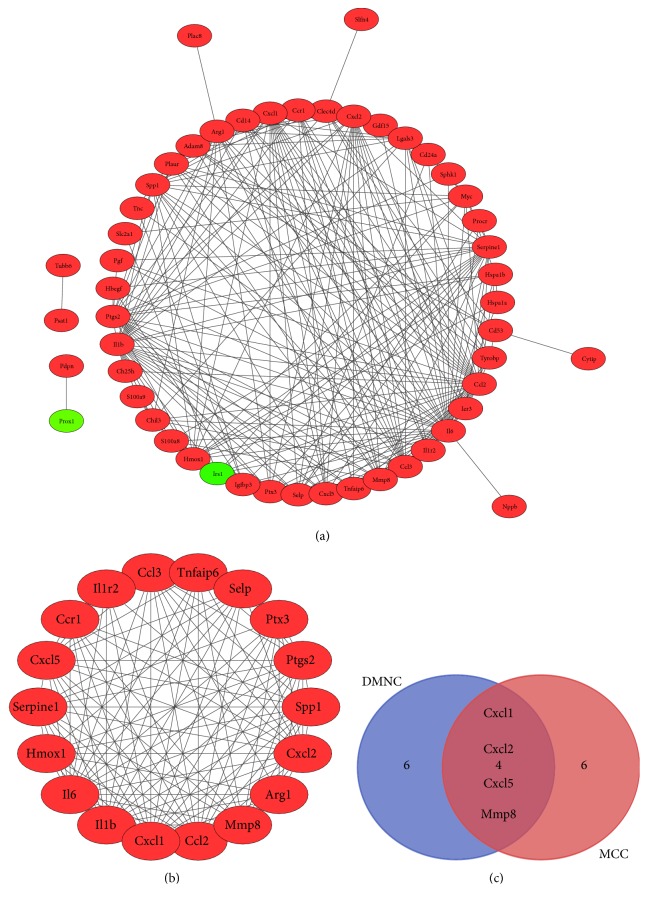
Establishment of PPI network, modules analysis, and Venn diagram. (a) Whole PPI network. Circles and lines represent genes and the interaction of proteins between genes, respectively. The red represents the upregulated genes. The green represents the downregulated genes. (b) PPI network of module. Circles and lines represent genes and the interaction of proteins between genes, respectively. The red represents the upregulated genes. (c) Venn diagram of mutual hub genes based on two methods. Abbreviation: PPI, protein-protein interaction.

In order to ulteriorly explore the biological characteristics of these DEGs, a PPI network was created using the STRING database. There were 56 nodes and 240 edges in this network, including 2 down- and 54 upregulated genes (see the supplementary document (available here)), as shown in Figure 6(a). Subsequently, a vital module was confirmed from the whole network, a total of 18 nodes and 117 edges in this module, as shown in Figure 6(b). 18 key genes in total were identified, including* Cxcl5, Arg1, Cxcl1, Spp1, Selp, Ptx3, Tnfaip6, Mmp8, Serpine1, Ptgs2, Il6, Il1r2, Il1b, Ccl3, Ccr1, Hmox1, Cxcl2, *and* Ccl2*.* Ccr1* was the most key gene in PPI network. These genes in the module were mainly enriched in the cytokine-cytokine receptor interaction, TNF signaling pathway, Toll-like receptor signaling pathway, and chemokine signaling pathway, as shown in Table 4. Utilizing the cytoHubba plug-in,* Cxcl1, Cxcl2, Cxcl5, *and* Mmp8* hub genes were screened out, as shown in Figure 6(c).

## 4. Discussion

AMI is one of the common kinds of coronary heart disease with high morbidity and mortality all over the world. In recent years, the number of patients with AMI is increasing annually. Controlling the number of patients with AMI and exploring the molecular mechanism of AMI are urgent to be solved.

In the study, using integrated bioinformatics and RRA analysis method, a total of 57 DEGs, including 2 down- and 55 upregulated genes, were identified from the GSE775, GSE19322, and GSE97494 database. From GO functional enrichment analysis, we identified that these DEGs were mainly enriched in the following functional categories, including receptor ligand activity, cytokine activity, cytokine receptor binding, G-protein coupled receptor binding, carbohydrate binding, chemokine activity, and chemokine receptor binding. Through KEGG pathway enrichment analysis, we found that the DEGs were chiefly enriched in the pathway of cytokine-cytokine receptor interaction, MAPK signaling pathway, TNF signaling pathway, Toll-like receptor signaling pathway, and chemokine signaling pathway. Utilising the STRING database, the PPI network was constructed. The module analysis filtered out 18 key genes, including* Cxcl5, Arg1, Cxcl1, Spp1, Selp, Ptx3, Tnfaip6, Mmp8, Serpine1, Ptgs2, Il6, Il1r2, Il1b, Ccl3, Ccr1, Hmox1, Cxcl2, *and* Ccl2*.* Ccr1* was the most fundamental gene in PPI network. Four hub genes in total were filtered out, including* Cxcl1, Cxcl2, Cxcl5, *and* Mmp8. *Most of these genes in AMI have been reported, which indicated that the results of integrated bioinformatics analysis were reliable.

Chemokine (C-C motif) receptor 1 (*Ccr1*), the highest score, was identified from the module.* Ccr1* is inflammation-associated gene, which may be a novel biomarker for the diagnosis and prognosis of AMI [13]. It exerts an important role in controlling inflammation [14]. Significantly, during the pathogenesis of AMI, inflammation of the coronary artery is the key process [15, 16]. We found that* Ccr1* mainly enriched in cytokine-cytokine receptor interaction and chemokine signaling pathway from the KEGG pathway analysis, which may be a direction of future research for diagnosis and treatment of AMI. In mice, chemokine (C-X-C motif) ligand 2 (*Cxcl2*) plays a kind of the potent neutrophil chemoattractants [17]. Using pharmacologic inhibition of circulating* Cxcl2*, researchers found neutrophil recruitment reduced at the site of myocardial infarction and injury within the infarcted myocardium alleviated [17]. Expression of* Cxcl2* and* Cxcl5* in AMI was elevated, which aggravated acute inflammation after myocardial injury and promoted cardiac rupture [18, 19]. From the KEGG pathway analysis, we found that* Cxcl2* mainly enriched in cytokine-cytokine receptor interaction, TNF signaling pathway, and chemokine signaling pathway. Thus,* Cxcl2* may play a key role in regulating cardiac remodeling following myocardial infarction (MI). Chemokine (C-C motif) ligand 3 (*Ccl3*) is also an important circulating chemokine. Tineke et al. [20] showed that* CCL3* is highly upregulated in patients with AMI. Vandervelde et al. clearly showed that the* Ccl3* mRNA expression was upregulated in ischemic myocardium [21]. These evidences indicated that* Ccl3* is closely associated with myocardial ischemia. Our study found that* Ccl3* was primarily enriched in cytokine-cytokine receptor interaction, Toll-like receptor signaling pathway, and chemokine signaling pathway. In experimental models of AMI, the innate immune response was induced through activation of Toll-like receptor (TLR)2 and TLR4 on circulating blood cells, which increases infarct size and influences ventricular remodeling [22, 23]. In the model of myocardial infarction, pharmacological inhibition of TLR2 or TRL4 can decrease monocyte inflow into the infarcted region, decrease the infarct area, and enhance myocardial remodeling [24–26]. From the above evidence, we identified the importance of cytokine-cytokine receptor interaction and chemokine signaling pathway in the occurrence and development of AMI.

Prostaglandin-endoperoxide synthase 2 (*PTGS2*, also named as COX-2), which can increase the neoplastic process by promoting proliferation, suppressing apoptosis, and angiogenesis, is an enzyme during conversion of arachidonic acid to prostaglandins [27].* PTGS2* has high expression in every kind of tumor, which was usually induced by cancer promoters, oncogenes, and cytokines [28].* PTGS2* gene associated with the decreasing risk of stroke and MI has been demonstrated [29]. Therefore, it plays a crucial role in treatment of MI. From KEGG pathway analysis, we found that* PTGS2* was enriched in TNF signaling pathway. It has been reported that TNF signaling pathway was associated with cardiac remodeling following MI [30]. So we speculate that* PTGS2 *exerts an important role on regulating cardiac remodeling following MI through TNF signaling pathway. We look forward to the result being confirmed by future experiments.

Among these genes, a novel gene* Tnfaip6 *(tumor necrosis factor-stimulated gene-6) was obviously differentially expressed in AMI. Interestingly, this gene was mainly reported in inflammatory bowel disease [31]. According to integrated bioinformatics analysis, we speculated that* Tnfaip6 *may play an important role in AMI, which could be a novel target for the treatment of AMI. Thus, further studies are needed in order to verify it.

Wei Gong et al. [32] have found that trimetazidine can prevent cardiac rupture in mice with AMI through inhibiting the expression of* Mmp2* and* Mmp9*, which indicates that the MMP family may be associated with cardiac remodeling after AMI. Matrix metalloproteinase-8 (*Mmp8*), a member of the MMP family, has gained growing attention in recent years. Previous research had only identified that types I, II, and III collagens are the substrates of* Mmp8*. However, in recent years, an increasing number of other proteins were detected as the substrates of* Mmp8*, including chemokine (C-X-C motif) ligand 5 (*CXCL5*) [33], macrophage inflammatory protein-1 [34], chemokine (C-X-C motif) ligand 11 (*CXCL11*) [35], and angiotensin-1 [36]. Research indicated that* Mmp8* can regulate the function and behavior of multiple cell types, including stem/progenitor cells [37], endothelial cells [38], smooth muscle cells [39], and neutrophils [34]. Study showed that gingival crevicular fluid* Mmp8 *concentrations significantly increase in patients with AMI [40]. Bioinformatics analysis indicates that* Mmp8* may be associated with prognosis of AMI. Nevertheless, little is known about the relation between* Mmp8* and cardiac remodeling. Therefore, more experiments were needed to verify it in the future.

It is noticeable that there have been papers researching the differentially expressed genes in AMI. However, the results of those papers were somewhat different from ours. The following reasons may account for this phenomenon: (1) some studies [3, 13], which have been reported, are peripheral blood microarray analysis of patients with AMI. Nevertheless, our study is microarray analysis of LV myocardium of mouse with AMI. Because the sample origin and the timing of specimen collection are different [4], which leads to somewhat different results, (2) different batches of microarray analysis, to some extent, also have somewhat different results; (3) compared with other studies of AMI, our study provides an integrated bioinformatics analysis of DEGs of AMI by means of statistical methods. We may provide credible results. Of course, it is important that the results are validated in follow-up experiments.

## 5. Conclusion

In conclusion, our study provides an integrated bioinformatics analysis of DEGs of AMI. This research provides numerous genes associated with AMI. This study may provide credible molecular biomarkers in terms of screening, diagnosis, and prognosis for AMI. Meanwhile, it also serves as a basis for exploring new therapeutic target for AMI. Compared with other studies of AMI, innovation point and merit of our current study was that the RRA method was utilized for the first time in exploring DEGs in AMI study. This study also has certain limitations. In this study, 18 microarrays were only screened, which is not enough. The limited sample size may easily lead to false positive results. Therefore, to verify the current findings, it is necessary to perform more experiments.

## Figures and Tables

**Table 1:** Details for GEO AMI data.

Reference	Sample	GEO	Platform	AMI	sham-operation
Schinke et al. (2003)	LV myocardium	GSE775	GPL81	3	3
Hunt et al. (2010)	LV myocardium	GSE19322	GPL339	4	4
Chikata et al. (2017)	LV myocardium	GSE97494	GPL6246	2	2

Abbreviation: GEO, Gene Expression Omnibus. AMI, acute myocardial infarction. LV, left ventricular.

**Table 2:** Screening DEGs in AMI by integrated microarray.

DEGs	Gene names
Upregulated	*Cxcl2,S100a8,Arg1,Il1r2,S100a9, Chil3,Slfn1,Slfn4,Ch25h,Serpine1,Il6,Ptx3,Cxcl1, Cxcl5,Tnc,Adam8,Hspa1b,Spp1,Hmox1,Hspa1a, Selp,Ankrd2,Cytip,Il1b,Ptgs2,Hbegf,Ccl3, Gadd45g,Pdpn,Cd53,Plaur,Myc,Aldh1a2,Plac8, Lgals3,Pgf,Gdf15,Ccl2,Mmp8,Ctla2b,Cd24a, Clec4d,Cd14,Slc2a1,Procr,Ifitm6,Igfbp3, Tnfaip6,Sphk1,Nppb,Tyrobp,Tubb6,Ccr1, Psat1,Ier3*
Downregulated	*Irs1,Prox1*

Abbreviation: DEGs, differentially expressed genes.

**Table 3:** GO analysis of genes associated with AMI.

Term	Description	Count	*P*-value
GO:0048018	receptor ligand activity	13	8.99E-11
GO:0005125	cytokine activity	9	2.63E-09
GO:0008009	chemokine activity	5	5.77E-08
GO:0005126	cytokine receptor binding	9	7.10E-08
GO:0042379	chemokine receptor binding	5	5.14E-07
GO:0045236	CXCR chemokine receptor binding	3	4.59E-06
GO:0008083	growth factor activity	5	2.57E-05
GO:0030246	carbohydrate binding	6	4.20E-05
GO:0001664	G-protein coupled receptor binding	6	6.03E-05
GO:0050542	icosanoid binding	2	0.000248517
GO:0050543	icosatetraenoic acid binding	2	0.000248517
GO:0070851	growth factor receptor binding	4	0.000399461
GO:0035325	Toll-like receptor binding	2	0.000428787
GO:0036041	long-chain fatty acid binding	2	0.000743064
GO:0070492	oligosaccharide binding	2	0.000743064
GO:0016209	antioxidant activity	3	0.000961581
GO:0005539	glycosaminoglycan binding	4	0.001232506
GO:0019956	chemokine binding	2	0.00149192
GO:0051787	misfolded protein binding	2	0.00149192
GO:0001530	lipopolysaccharide binding	2	0.001619175
GO:0031406	carboxylic acid binding	4	0.001701432
GO:0043177	organic acid binding	4	0.001850987
GO:0019955	cytokine binding	3	0.002141304
GO:0001968	fibronectin binding	2	0.002329934
GO:1901567	fatty acid derivative binding	2	0.002815131
GO:0044183	protein binding involved in protein folding	2	0.003914772
GO:0031072	heat shock protein binding	3	0.004127547
GO:0005504	fatty acid binding	2	0.004741625
GO:0008201	heparin binding	3	0.005037273
GO:0048020	CCR chemokine receptor binding	2	0.005409964

**Table 4:** Top 20 KEGG pathway enrichment analyses of DEGs associated with AMI.

pathway	ID	Gene count	*p*-value	adjust *p*-value	Genes
IL-17 signaling pathway	mmu04657	9	2.32E-10	3.30E-08	*Cxcl2,S100a8,S100a9,Il6,Cxcl1,Cxcl5,Il1b,Ptgs2,Ccl2*
Legionellosis	mmu05134	7	6.85E-09	4.87E-07	*Cxcl2,Il6,Cxcl1,Hspa1b,Hspa1a,Il1b,Cd14*
Cytokine-cytokine receptor interaction	mmu04060	10	6.80E-07	3.22E-05	*Cxcl2,Il1r2,Il6,Cxcl1,Cxcl5,Il1b,Ccl3,Gdf15,Ccl2,Ccr1*
Salmonella infection	mmu05132	6	1.38E-06	4.88E-05	*Cxcl2,Il6,Cxcl1,Il1b,Ccl3,Cd14*
TNF signaling pathway	mmu04668	6	1.03E-05	0.000292216	*Cxcl2,Il6,Cxcl1,Il1b,Ptgs2,Ccl2*
Rheumatoid arthritis	mmu05323	5	3.97E-05	0.00093865	*Il6,Cxcl5,Il1b,Ccl3,Ccl2*
Hematopoietic cell lineage	mmu04640	5	7.17E-05	0.001297709	*Il1r2,Il6,Il1b,Cd24a,Cd14*
Malaria	mmu05144	4	7.31E-05	0.001297709	*Il6,Selp,Il1b,Ccl2*
Toll-like receptor signaling pathway	mmu04620	5	8.73E-05	0.001377696	*Il6,Spp1,Il1b,Ccl3,Cd14*
Chagas disease (American trypanosomiasis)	mmu05142	5	0.000105431	0.001497126	*Serpine1,Il6,Il1b,Ccl3,Ccl2*
Amoebiasis	mmu05146	5	0.000120813	0.001559588	*Arg1,Il1r2,Il6,Il1b,Cd14*
Human cytomegalovirus infection	mmu05163	7	0.000153378	0.00181497	*Il6,Il1b,Ptgs2,Ccl3,Myc,Ccl2,Ccr1*
Transcriptional misregulation in cancer	mmu05202	6	0.000179856	0.001964578	*Il1r2,Il6,Gadd45g,Myc,Cd14,Igfbp3*
Chemokine signaling pathway	mmu04062	6	0.000291266	0.002954267	*Cxcl2,Cxcl1,Cxcl5,Ccl3,Ccl2,Ccr1*
MAPK signaling pathway	mmu04010	7	0.000358914	0.003397722	*Hspa1b,Hspa1a,Il1b,Gadd45g,Myc,Pgf,Cd14*
Pertussis	mmu05133	4	0.000404457	0.003589552	*Il6,Cxcl5,Il1b,Cd14*
Kaposi sarcoma-associated herpesvirus infection	mmu05167	6	0.000451092	0.003767942	*Cxcl2,Il6,Cxcl1,Ptgs2,Myc,Ccr1*
Prion diseases	mmu05020	3	0.00050479	0.003982228	*Il6,Hspa1a,Il1b*
AGE-RAGE signaling pathway in diabetic complications	mmu04933	4	0.001183968	0.008848603	*Serpine1,Il6,Il1b,Ccl2*
HIF-1 signaling pathway	mmu04066	4	0.001367781	0.009711245	Serpine1,Il6,Hmox1,Slc2a1

## Data Availability

The data used to support the findings of this study are included within the supplementary information file.

## Supplementary Materials

Supplementary Materials A PPI network: there were 56 nodes and 240 edges in this network, including 2 down- and 54 upregulated genes (see the supplementary document).
