# Supplementary figures and images for: Identification of Differentially Expressed Genes and Signaling Pathways in Acute Myocardial Infarction Based on Integrated Bioinformatics Analysis
Source: Cardiovasc Ther. 2019 Aug 1;2019:8490707. doi: 10.1155/2019/8490707 (PMC6739802; doi:10.1155/2019/8490707)

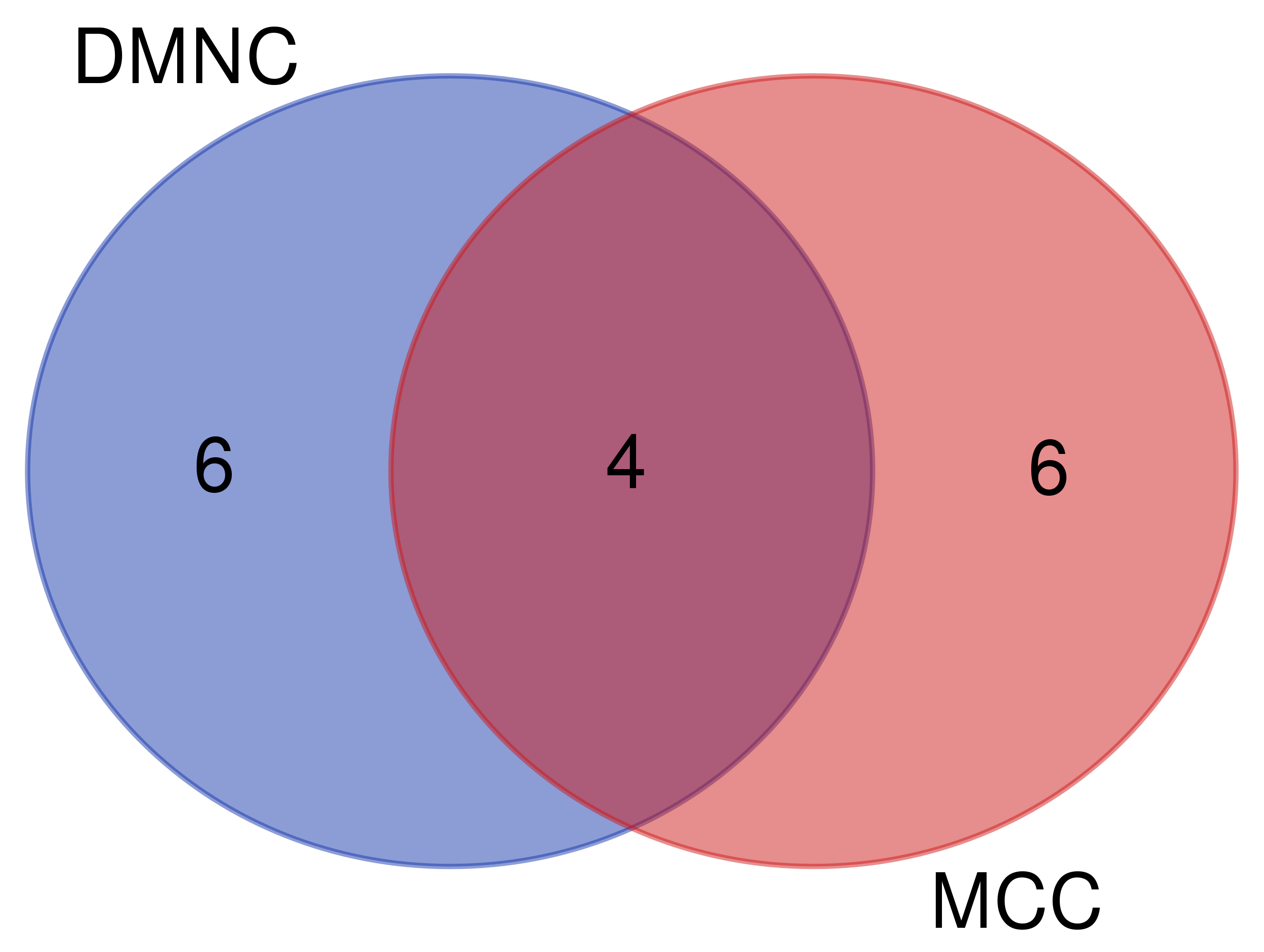

Supplement: Supplementary Materials — A PPI network: there were 56 nodes and 240 edges in this network, including 2 down- and 54 upregulated genes (see the supplementary document). [file 8490707.f1.zip › DMNC and MCC/╬ñ╢≈═╝.png]

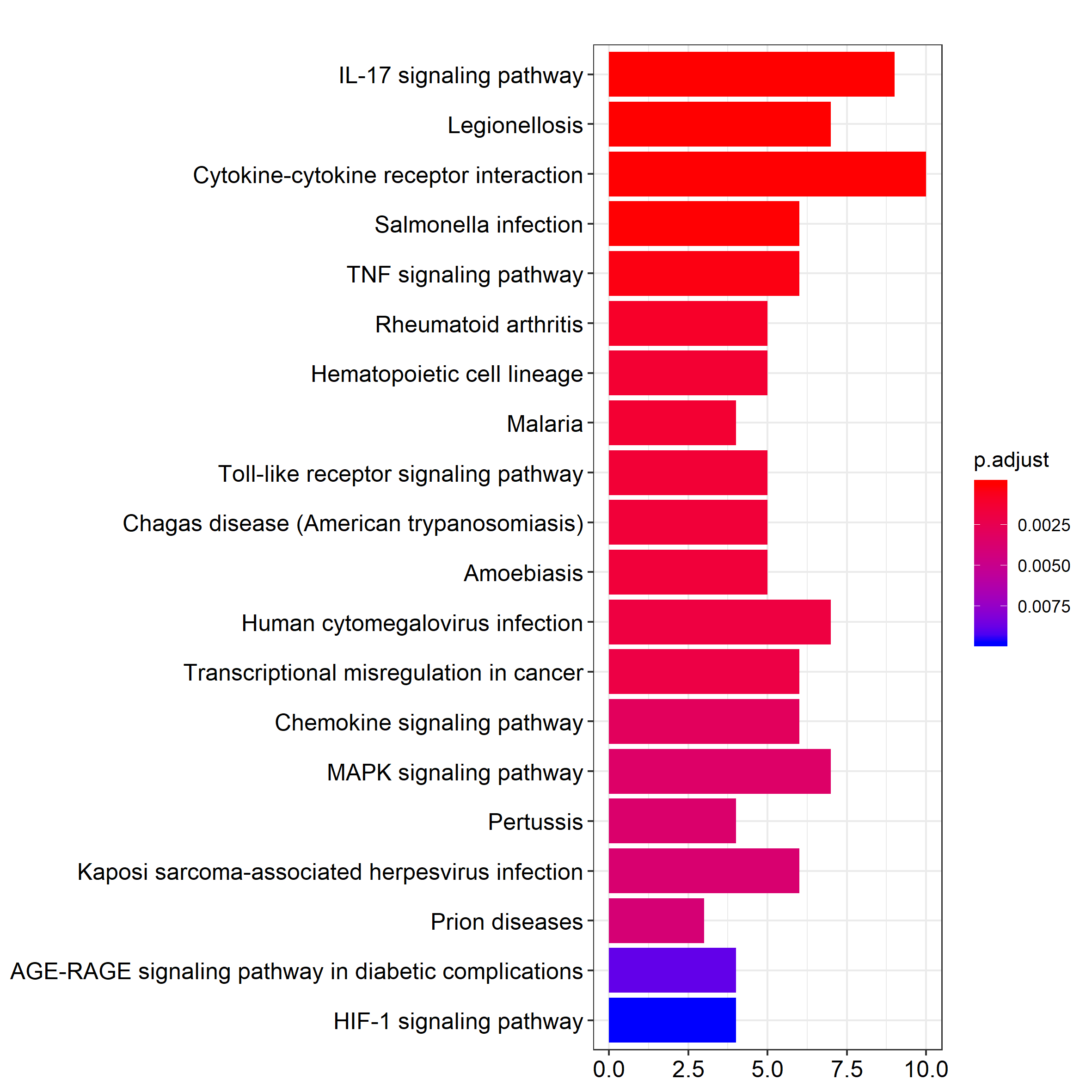

Supplement: Supplementary Materials — A PPI network: there were 56 nodes and 240 edges in this network, including 2 down- and 54 upregulated genes (see the supplementary document). [file 8490707.f1.zip › GO and KEGG/barplot-KEGG.tiff]

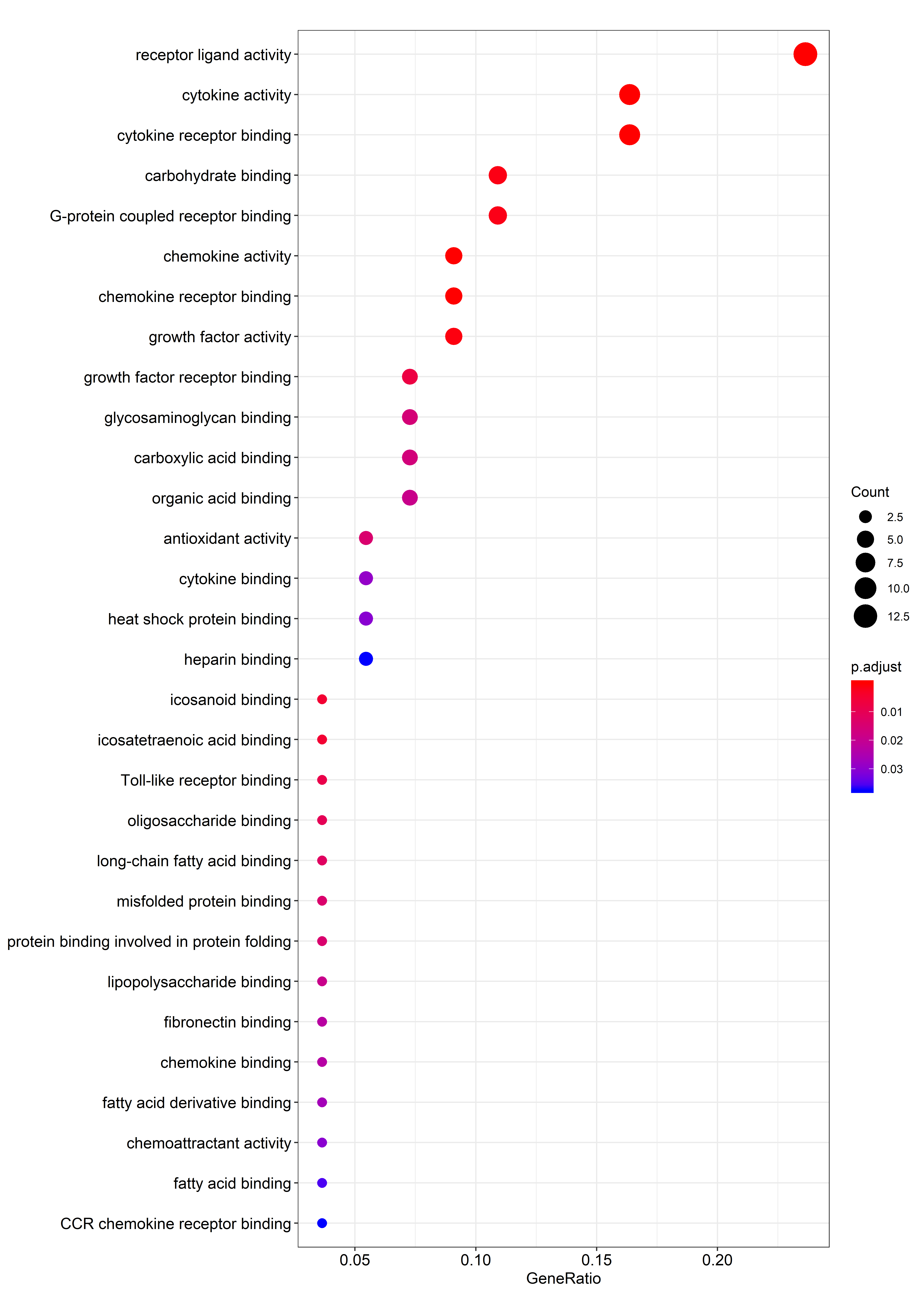

Supplement: Supplementary Materials — A PPI network: there were 56 nodes and 240 edges in this network, including 2 down- and 54 upregulated genes (see the supplementary document). [file 8490707.f1.zip › GO and KEGG/dotplot GO.tiff]

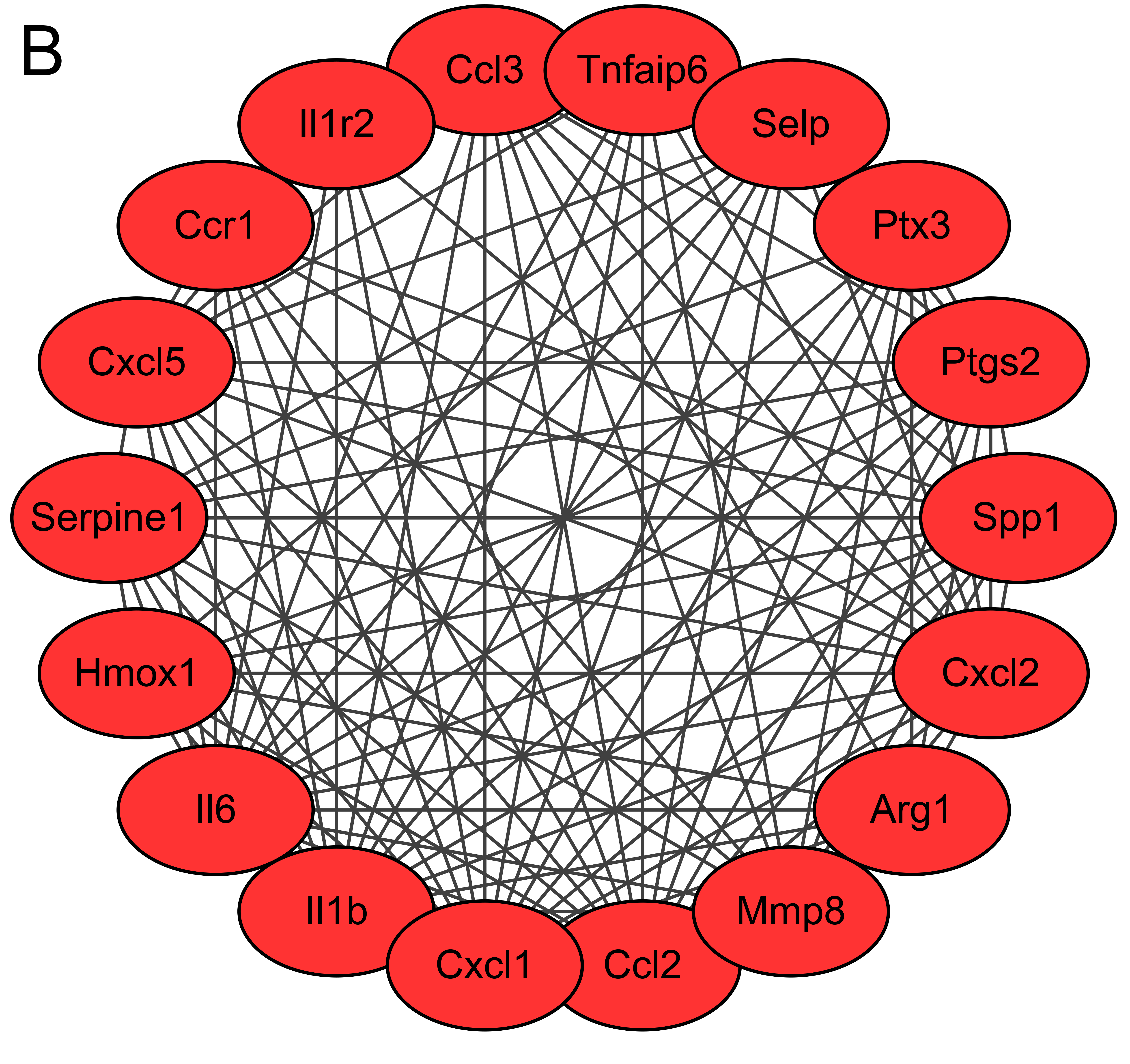

Supplement: Supplementary Materials — A PPI network: there were 56 nodes and 240 edges in this network, including 2 down- and 54 upregulated genes (see the supplementary document). [file 8490707.f1.zip › PPI network of module/module-1.tif]

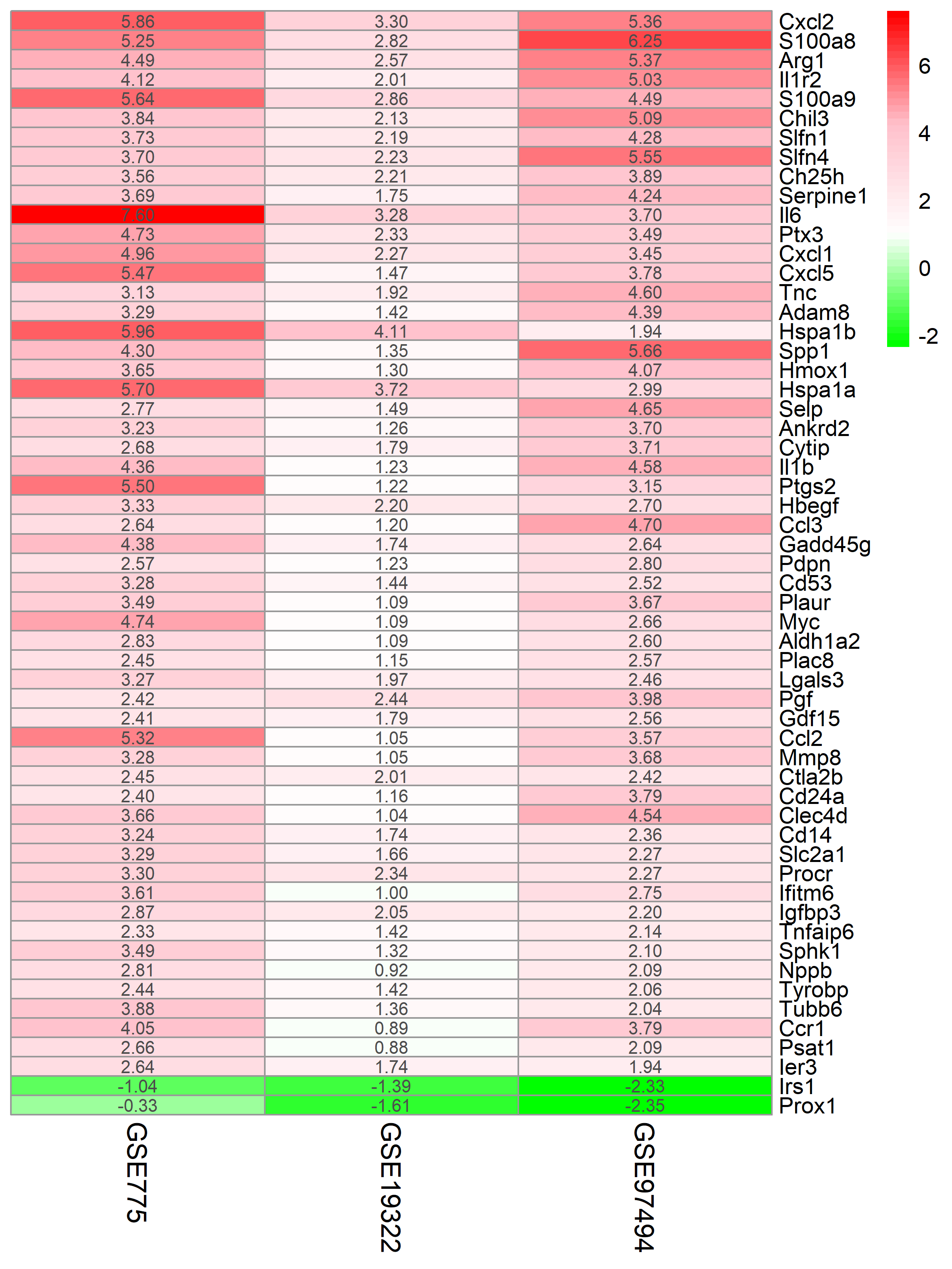

Supplement: Supplementary Materials — A PPI network: there were 56 nodes and 240 edges in this network, including 2 down- and 54 upregulated genes (see the supplementary document). [file 8490707.f1.zip › The heatmap of three gene expression microarrays by utilizing the RRA approach/logFC5.tiff]

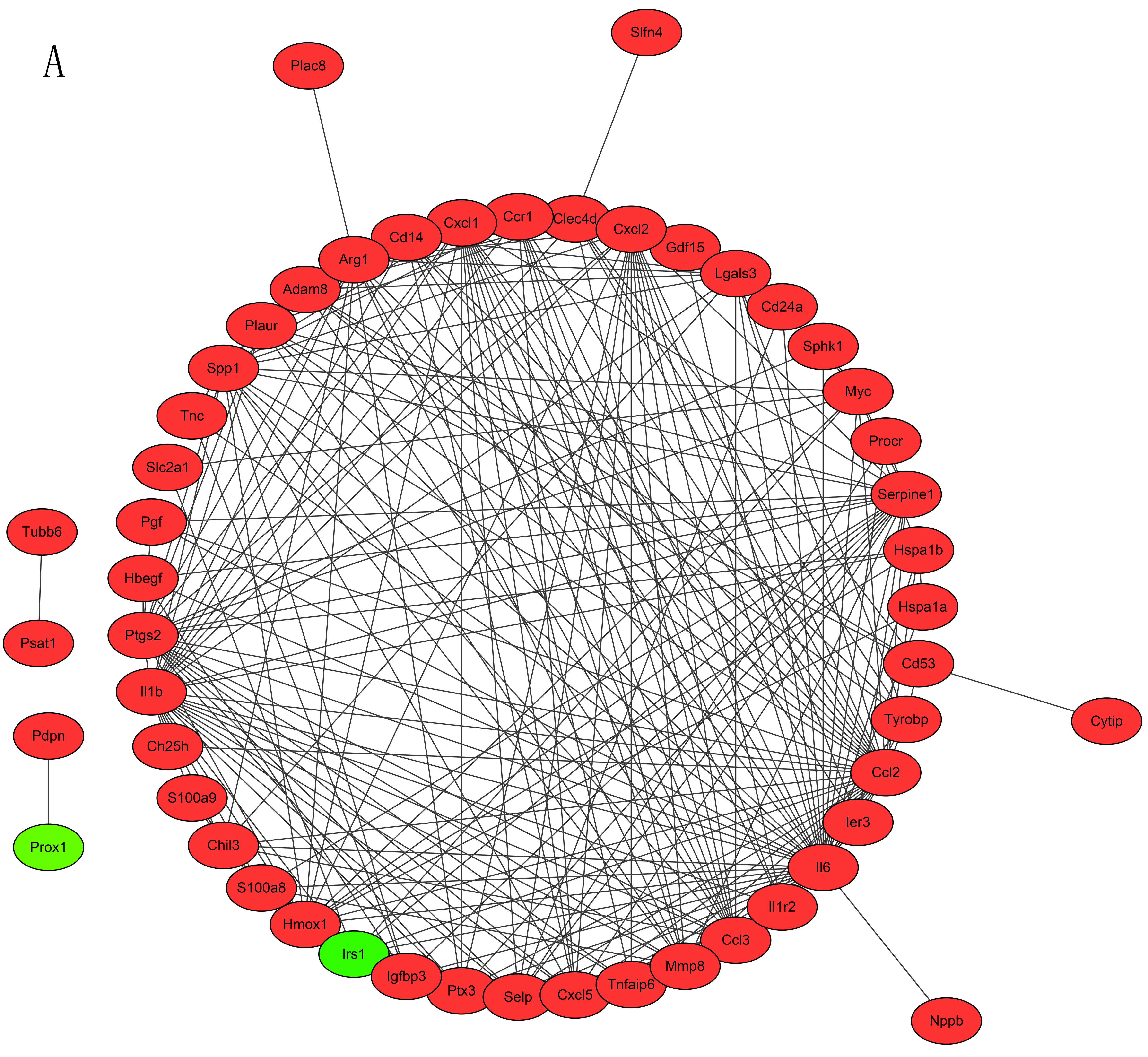

Supplement: Supplementary Materials — A PPI network: there were 56 nodes and 240 edges in this network, including 2 down- and 54 upregulated genes (see the supplementary document). [file 8490707.f1.zip › Whole PPI network/Figure 6A.tif]
